# Focusing on the hypoxia-inducible factor pathway: role, regulation, and therapy for osteoarthritis

**DOI:** 10.1186/s40001-022-00926-2

**Published:** 2022-12-12

**Authors:** Hanhan Guo, Jianghong Huang, Yujie Liang, Daping Wang, Huawei Zhang

**Affiliations:** 1 Department of Biomedical Engineering, Southern University of Science and Technology, Shenzhen, 518055 China; 2 Department of Spine Surgery and Orthopedics, Shenzhen Second People’s Hospital (First Affiliated Hospital of Shenzhen University, Health Science Center), Shenzhen, 518035 China; 3 Innovation Leading Engineering Doctor, Tsinghua University Shenzhen International Graduate School, Class 9 of 2020, Shenzhen, 518055 China; 4 Department of Child and Adolescent Psychiatry, Shenzhen Kangning Hospital, Shenzhen Mental Health Center, Shenzhen, 518020 China; 5 Department of Orthopedics, Shenzhen Intelligent Orthopaedics and Biomedical Innovation Platform, Guangdong Provincial Research Center for Artificial Intelligence and Digital Orthopedic Technology, Shenzhen Second People’s Hospital, The First Affiliated Hospital of Shenzhen University, Shenzhen, 518000 China; 6 Guangdong Provincial Key Laboratory of Advanced Biomaterials, Southern University of Science and Technology, Shenzhen, 518055 China

## Abstract

Osteoarthritis (OA) is a common chronic disabling disease that affects hundreds of millions of people around the world. The most important pathological feature is the rupture and loss of articular cartilage, and the characteristics of avascular joint tissues lead to limited repair ability. Currently, there is no effective treatment to prevent cartilage degeneration. Studies on the mechanism of cartilage metabolism revealed that hypoxia-inducible factors (HIFs) are key regulatory genes that maintain the balance of cartilage catabolism−matrix anabolism and are considered to be the major OA regulator and promising OA treatment target. Although the exact mechanism of HIFs in OA needs to be further clarified, many drugs that directly or indirectly act on HIF signaling pathways have been confirmed by animal experiments and regarded as promising treatments for OA. Targeting HIFs will provide a promising strategy for the development of new OA drugs. This article reviews the regulation of HIFs on intra-articular cartilage homeostasis and its influence on the progression of osteoarthritis and summarizes the recent advances in OA therapies targeting the HIF system.

## Introduction

In the recent years, as one of the most common chronic diseases of orthopedics, the incidence of osteoarthritis has been increasing year by year with the aging of the population and the increasing proportion of obesity [1]. The most important change in this disease is the destruction of articular cartilage, which is mainly caused by the decomposition of extracellular matrix by degradation enzymes and the death of chondrocytes caused by apoptosis or autophagy. Owing to the lack of blood supply and the relatively closed joint cavity, the articular cartilage itself is in a hypoxic environment. Since there are no capillaries in articular cartilage, the oxygen concentration gradient varies from only 1–10% [2]. The physiological homeostasis of this hypoxic environment is mainly regulated by hypoxia-inducible factors (HIFs), especially HIF-1 and HIF-2 [3]. The hypoxic environment induces chondrocytes to produce a series of hypoxia-related molecules, which are involved in the regulation of osteoarthritis extracellular matrix-degrading enzymes and chondrocyte autophagy and apoptosis. The purpose of this article is to review recent studies on the HIF signaling pathway and its roles in the occurrence and development of osteoarthritis and to explore potential therapies targeting the HIF system.

## HIF family

**Fig. 1:**
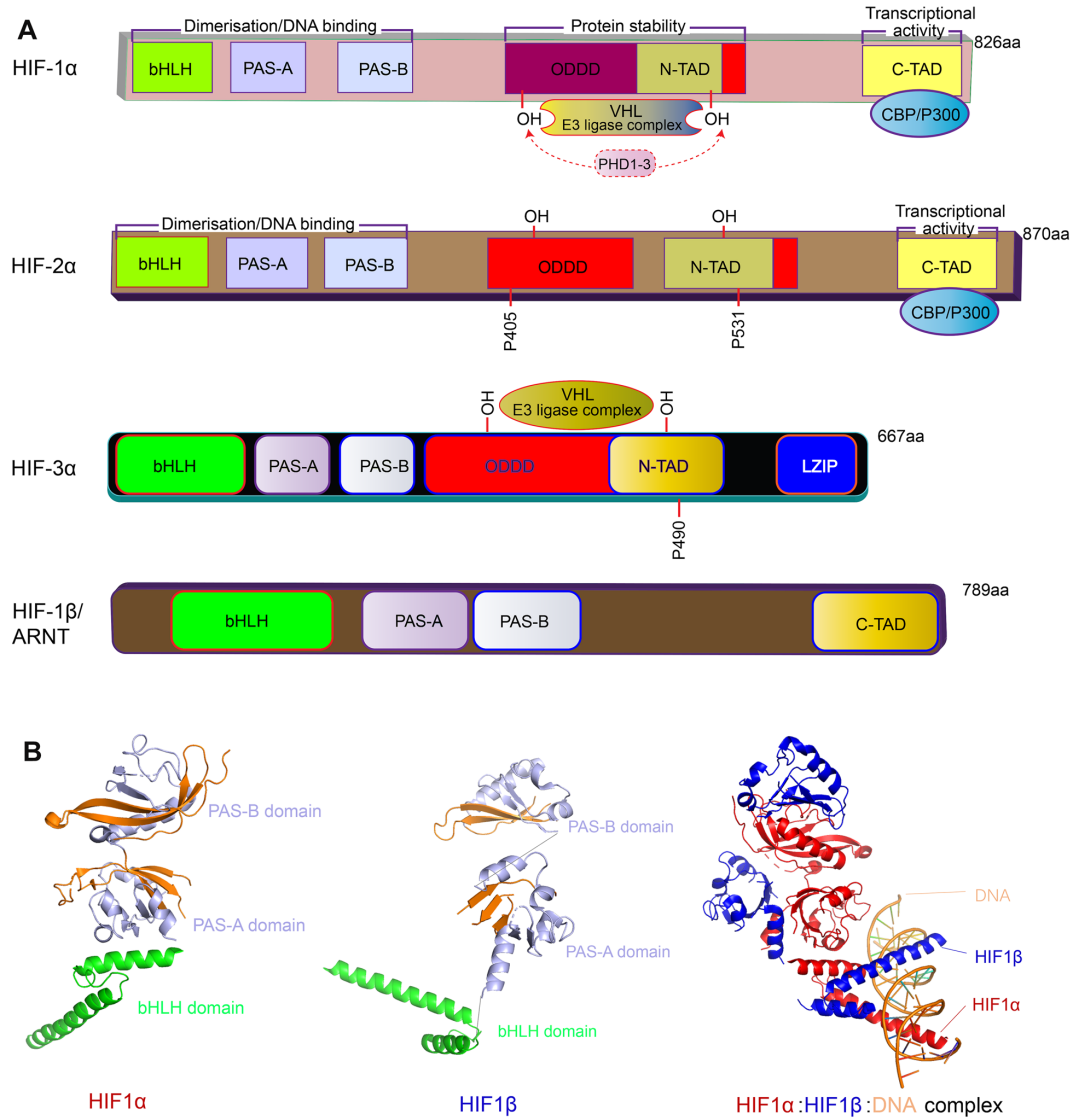
Schematic diagram of the domain organization of HIFs. **A** Illustrated functional domain arrangements of HIFs. Domains: bHLH, basic helix-loop-helix (DNA binding and dimerization); PAS, Per/Ahr-ARNT/Sim (dimerization); PAS-A PAS-associated domain A; PAS-B PAS-associated domain B; ODDD: oxygen-dependent degradation domain; N-TAD, N-terminal transactivation domain (transcriptional transactivation); C-TAD, C-terminal transactivation domain (transcriptional transactivation); Factors: PHD1-3, HIF prolyl hydroxylase 1–3; VHL, von Hippel–Lindau tumor suppressor protein. CBP/P300: p300/CREB-binding protein. **B** 3D structural illustrations of HIF domain organization. Mouse HIF-1α:HIF-1β:HRE (DNA element) are shown as an example to show domain organizations. The structure is retrieved from the Protein Data Bank (ID: 4ZPR). Color schemes are the same as in panel **A**. Because the structure only contains partial sequences of HIF1α:HIF-1β, only bHLH and PAS domains are shown

HIFs are heterodimeric transcription factors composed of α (HIF-1α, HIF-2α, and HIF-3α) and β (HIF-1β, HIF-2β, and HIF-3β, also known as ARNT1, ARNT2, and ARNT3) subunits [4–6]. HIF-α and HIF-β have the same structural characteristics: both possess basic helix-loop-helix (bHLH) and PAS domains (PAS is named after the three proteins PER, ARNT and SIM all of which have this domain) [7] (Fig. 1A, B). The bHLH-PAS domains mediate heterodimerization and further bind with the hypoxia response elements (HREs) of the target genes [8]. Binding of HIF-1α to HRE causes upregulation of HIF-1α target genes and is precisely regulated by many factors [9, 10]. Besides bHLH and PAS domains, the HIF-1α protein also contains an oxygen-dependent degradation (ODD) domain and two transactivation domains (N-TAD and C-TAD). Interestingly, HIF-1β only has the C-terminal transactivation domain (C-TAD), however, it has two repeat regions PAS domains known as PAS-A and PAS-B. HIF-2β and HIF-3β have similar domain organizations as HIF-1β (Fig. 1A, B).

Similar to HIF-1α, HIF-2α also interacts with HREs to upregulate transcriptional activity of target genes [11]. The HIF-2α protein shares 48% sequence identity with HIF-1α protein, and has many structural and biochemical similarities with HIF-1α (e.g., heterodimerization and HREs binding). However, when compared with HIF-1α, which is widely expressed, HIF-2α is mainly expressed in the lung, carotid body, and endothelial cells [12]. HIF-2β and HIF-3β also expression in the endothelial tissue and have 70% similarity with HIF-1β and sharing similar structure [13–15]. In contrast, HIF-3α is involved in hypoxic downregulation through selective splicing of transcription factors that may act as inhibitors of HIF-1α [16]. HIF-3α is also expressed in a variety of tissues, dimerizes with HIF-1α and binds to HREs [17]. Currently, HIF-1 and HIF-2 have been more widely studied, while HIF-3 and other HIFs have been relatively less studied (Fig. 1A, B).

## Regulation of HIFs

HIFs are key heterodimer transcription factors expressed under hypoxic conditions. It mediates adaptive responses from normoxic (~ 21% oxygen) to hypoxic conditions by binding to the promoters of numerous hypoxia-inducible genes, such as those involved in iron metabolism, angiogenesis, and glucose metabolism. It plays important roles for cells and tissues to adapt to low oxygen tension [6, 18, 19].

The regulation of HIF system is mainly through the α subunit HIF-α, whereas β subunit HIF-β is constitutively expressed [20]. Under normoxic condition, the expression of two main HIF-α isoforms (HIF-1α and HIF-2α) is regulated by oxygen-independent mechanisms, the mitogen-activated protein kinase (MAPK) pathway and the growth factor-mediated phosphoinositide 3 kinase (PI3K) pathway [21, 22]. Taking the oxygen-independent mechanism as an example, HIF-α protein degradation is mediated by the ODD domain. HIF-α remains stable even in the absence of a hypoxia signal when the entire ODD region is removed [23]. Hydroxylation of proline residues 402 and 564 in the ODD domain controls the interaction between HIF-α and the von Hippel-Lindau tumor suppressor protein (pVHL) which facilitates the ubiquitination and degradation of HIIF-α [24–27]. The hydroxylation process is regulated by three conserved HIF prolyl hydroxylases (PHD1, PHD2, PHD3, and also known as EGLN2, EGLN1, EGLN3), and their activity lies in the presence of oxygen, iron, 2-oxoglutarate and ascorbate [18, 28]. Interestingly, it has been shown that PHD2 has remarkable significance in regulating HIF-1α levels by using small interfering RNA (siRNA) techniques [29].

**Fig. 2:**
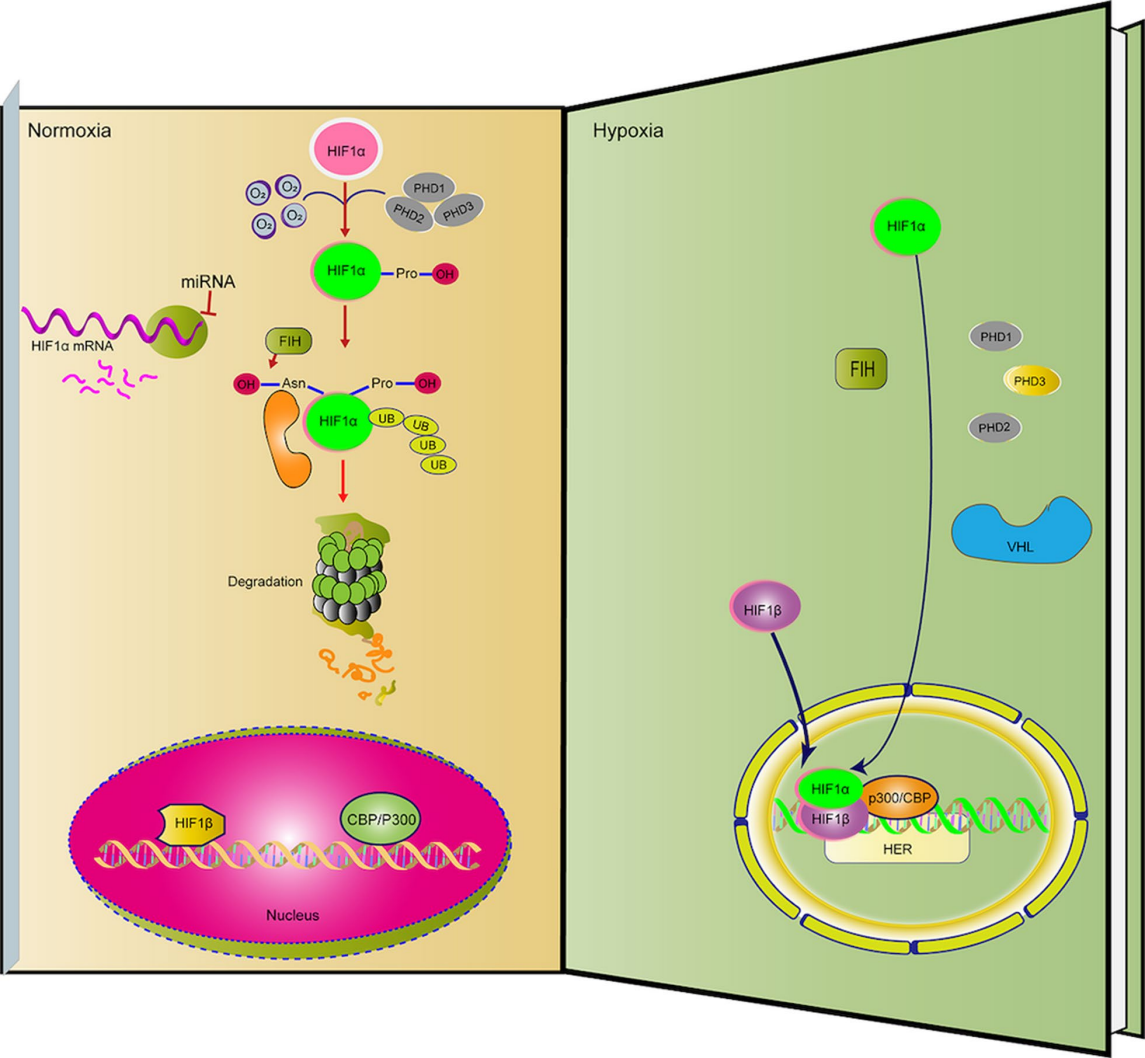
Schematic diagram of the HIF-1 pathway. Under normal oxygen conditions, HIF-1α protein is hydroxylated by prolyl 4-hydroxylase (PHD) on proline residues and polyubiquitinated by von Hippel–Lindau protein (pVHL). In this case, it will be degraded by the 26S proteasome system. Under hypoxia, HIF-1α can enter the nucleus and form a transcription complex with HIF-1β subunits and then recruit coactivators (such as CBP/p300) to regulate the transcriptional activity of downstream genes. PHD, molecules containing proline-hydroxylase domain; Ub, ubiquitin; FIH, a novel protein that interacts with HIF-1alpha and VHL to mediate repression of HIF-1 transcriptional activity

In hypoxia, prolyl hydroxylation of the ODD domain is suppressed, and the interaction between HIF-1α and pVHL is inhibited. As a result, HIF-α degradation is interrupted and its concentration consequently increases. HIF-α is then translocated and accumulated in the nucleus where it binds to HIF-β via bHLHs and PAS domains to form the HIF-α/β dimer complex [6]. Transcriptional coactivators, such as p300/CBP (p300/CREB-binding protein), help the HIF complex couple to HRE elements within the promoter region of HIF target genes, consequently regulating their transcriptional activation [30, 31]. Asparagine hydroxylase, known as FIH-1 (factor inhibiting HIF-1), also mediates the transcriptional activity of HIF-1α [32–35]. Hydroxylation of Asn803 from FIH-1 blocks the HIF-1α interaction with p300/CBP in its C-TAD under normoxic conditions, so FIH-1 acts as a negative regulator of HIF-1α by interacting with pVHL to suppress transcriptional activity and modulate stabilization (Fig. 2).

## The roles of HIF-1α in OA

It is extremely important to maintain chondrocyte metabolism under hypoxic conditions. In healthy cartilage, hypoxic conditions can enhance the expression and activity of HIF-1α [36–38], which not only induces the expression of Erythropoietin (EPO), SRY-Box Transcription Factor 9 (SOX9), Collagen Type II Alpha 1 (COL2A1), Vascular endothelial growth factor (VEGF), Nitric Oxide Synthase (NOS) and glucose transporter protein type 1 (GLUT1) to maintain cartilage homeostasis, but also inhibits the expression of Collagen Type I Alpha 1 (COL1A1), Collagen Type I Alpha 2 (COL1A2) and Collagen Type III Alpha 1 (COL3A1) to prevent the degradation of the extracellular matrix (ECM) [39–42], thereby mediating anti-catabolic reactions and preventing spontaneous and induced destruction of human cartilage. In addition, HIF-1α can also protect chondrocytes from apoptosis by inducing heat shock protein 70 (HSP70) to increase ECM gene expression levels and cell viability [43–50].

Studies have found that HIF-1α levels are significantly related to the severity and progression of osteoarthritis [51, 52]. In the early stage of OA, articular cartilage undergoes metabolic adaptation under harmful stimulating conditions. Chondrocytes dedifferentiate into a hypertrophic phenotype, and ECM is degraded which is characterized by decreased synthesis of Collagen II and newly synthesized Collagen I and Collagen X, accompanied by activation of matrix-degrading enzymes such as MMP13 [53]. During this period, chondrocytes attemp to repair damaged cells and ECM through adaptive changes in metabolism. Owing to the changes in the chondrocyte microenvironment in OA, the activation of AMP-activated protein kinase (AMPK) and inhibition of the mammalian target of rapamycin (mTOR) induced by HIF-1α can also cause chondrocyte autophagy [54]. Osteoarthritis of the temporomandibular joint (TMJ) is one of the common types of OA. In TMJ-OA, the HIF-1-VEGF-Notch signaling pathway accelerates cartilage angiogenesis, thereby accelerating the development of TMJ-OA [55–57]. F-box and WD repeat domain-containing 7 (FBW7) can negatively regulate the HIF-1α/VEGF pathway to inhibit angiogenesis, thereby inhibiting the degradation of chondrocytes induced by IL-1β [58]. Studies have found that the expression of HIF-1α and Runx2 in degenerative chondrocytes increases simultaneously. Studies showed that Runx2 protein can induce the expression of HIF-1α at the transcriptional level and accelerate the progression of OA [59]. However, with the development of OA, this metabolic adaptation and the self-repair ability of cartilage decrease, leading to serious tissue damage [60, 61]. The abnormal deposition of ECM can further lead to synovial fibrosis. Among them, TGF-β, procollagen-lysine, 2-oxoglutarate 5-dioxygenase2, COL1A1 and tissue inhibitor of metalloproteinases 1 are involved in osteoarthritis-related fibrosis and are considered fibrosis markers [62–64]. Fibroblast-like synovial cells (FLSs) are the main effector cells of synovial fibrosis in knee osteoarthritis [65]. More recently, a form of programmed inflammatory cell death called pyroptosis has been discovered [66]. HIF-1α activates nucleotide-binding oligomerization domain-like receptor family pyrin domain-containing 3 (NLRP3), assembles inflammasome complexes through the adapter protein apoptosis-associated speck-like protein containing a caspase recruitment domain, and drives caspase-1-mediated inflammation to mediate knee synovial fibrosis in OA [67–69]. The HIF-1α/NLRP3 inflammasome is one of the inflammasome signal transductions closely related to the KOA process. Inhibiting the activation of inflammasomes improves synovial fibrosis in KOA [70–73].

In addition, the progression of HIF-1α in OA is also regulated by long noncoding RNAs (lncRNAs) and miRNAs. LncRNAs refer to a subpopulation of noncoding RNAs longer than 200 nucleotides. Previous studies have shown that abnormal expression of lncRNAs plays an important role in the development of OA [74, 75]. For example, lncRNA UFC1 can increase the proliferation of chondrocytes in OA [76], and lncRNA cartilage injury-related (lncRNA-CIR) can promote the degradation of chondrocyte extracellular matrix in the disease [77]. Long noncoding HIF-1α co-activating RNA (LncHIFCAR) positively regulates HIF-1α and HIF-1α target genes (such as VEGF and BNIP3), thereby promoting the hypoxia-induced inflammatory response and matrix synthesis and inducing cell apoptosis [78]. miRNAs are single-stranded small noncoding RNAs abundant in cells. Many miRNAs also plays an important role in hypoxia cartilage homeostasis or the OA stress microenvironment through the HIF-1α pathway [79, 80]. For example, miR-146a upregulates the expression of ULK-1, HIF-1α and ATG-5 by targeting TRAF6/IRAK1, thereby slowing the progression of OA [81]. miR-204 and miR-211 affect nerve growth factor (NGF) expression in a Runx2-dependent manner to regulate homeostasis and OA progression [82]. miR-411 regulates chondrocyte autophagy by targeting HIF-1α [83]. Whereas miR-373 regulates the damage to chondrocytes treated with lipopolysaccharide by targeting HIF-1α [84].

## The roles of HIF-2α in OA

HIF-2α and HIF-1α have different functions in cartilage. In the articular cartilage cells of synovial joints, HIF-1α promotes the homeostatic pathway, while HIF-2α promotes the degradation pathway. HIF-2α can target genes related to the hypertrophy and differentiation of chondrocytes, such as Runt-related transcription factor 2 (RUNX2) and COL10A1, and genes related to the degradation of ECM, such as matrix metalloproteinase MMP9, MMP13, MMP3 and A Disintegrin and Metalloproteinase with Thrombospondin motifs 48–51 (ADAMTS48-51) [85]. In addition, proinflammatory factors (such as interleukin IL-1β, IL-6, and tumor necrosis factor TNF-α) can upregulate the expression of HIF-2α in articular chondrocytes by activating Nuclear factor kappa-light-chain-enhancer of activated B cells (NF-κB) signaling pathways [86, 87], thereby promoting chondrocytes from the prehypertrophic state to the terminal hypertrophic state [87]. Previous studies have found that nicotinamide phosphoribosyltransferase (NAMPT) is the direct target gene of HIF-2α in articular chondrocytes and is upregulated in OA cartilage. Inhibition of NAMPT enzyme activity by injection of the NAMPT inhibitor FK866 (intra-articular or intraperitoneal) can inhibit the destruction of osteoarthritic cartilage caused by intra-articular injection or DMM surgery with Epas1 adenovirus (Ad-Epas1) or Ad-Nampt [88]. Fas (CD95) is a member of the tumor necrosis factor receptor family, containing a death domain to activate apoptosis signals. The binding of Fas ligand (FasL) or an agonistic anti-Fas antibody to the Fas receptor triggers the apoptosis signal. The increase in the chondrocyte apoptosis is also related to the severity of human OA cartilage damage [89, 90]. The combination of FasL or anti-Fas antibodies can induce chondrocyte apoptosis [91, 92]. Researchers have found that HIF-2α promotes Fas-mediated chondrocyte apoptosis by upregulating Fas expression [93]. The accumulation of iron-dependent lipid hydroperoxides causes cell death, known as ferroptosis [94–96]. The recent evidence suggests that ferroptosis in chondrocytes is associated with the progression of OA [94–96]. These findings indicate that ferroptosis suppression is a new potential choice to prevent the progression of OA. Human lysyl oxidase (LOX) is a hypoxia response gene whose product can catalyze collagen cross-linking, while HIF-2α can upregulate LOX and play a crucial role in osteoarthritis [97, 98]. HIF-2α also causes cartilage destruction by regulating the expression of various catabolic factors, such as VEGF, type X collagen, prostaglandin intra peroxidase synthase 2 (PTGS2) and nitric oxide synthase 2 (NOS2) [87].

**Fig. 3:**
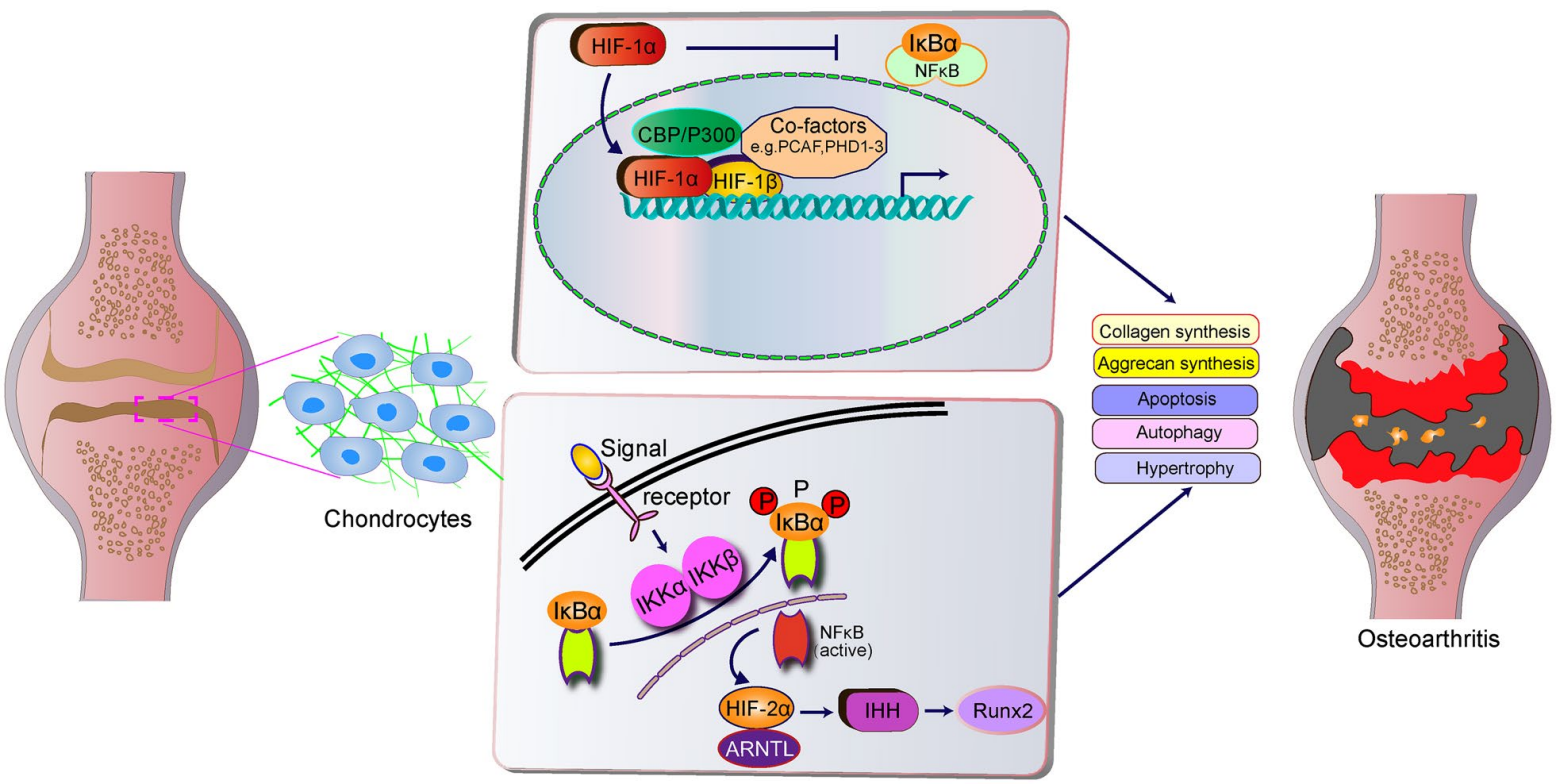
HIF-1α and HIF-2α have different functions in OA. HIF-1α mainly maintains the extracellular matrix synthesis of chondrocytes and chondrocyte differentiation and promotes the balance of articular cartilage autophagy in the body. NF-κB activation promotes the heterodimerization of HIF-2α and RNTL, leading to the activation of the transcription factor HIF-2α, and through the IHH and RUNX2 axes, coactivating MMP13 prompts articular chondrocytes to show a hypertrophic state of differentiation, leading to the occurrence and progression of OA

When compared with normal cartilage, the expression of various miRNAs in osteoarthritis has also undergone some changes, which indicates that the expression of miRNAs may also be involved in the metabolic balance of cartilage through HIF-2α pathway [99]. There are experiments have shown that miR-365 regulates HIF-2α at the posttranscriptional level and cross-regulates the MAPK-NF-kB signaling pathway to reduce IL-1β-induced chondrocyte catabolism [100] (Fig. 3).

## Targeting HIF-1α for OA therapy

Because PHD can hydroxylate HIF-1α and lead to ubiquitination degradation, inhibiting the hydroxylation of HIF-1α by PHD may have potential therapeutic value. Dimethyloxaloylglycine (DMOG), an analog of 2-oxoglutarate, can competitively bind to PHD and eventually inhibit HIF degradation [101]. In addition, the PHD inhibitors TM6008 and TM6089 are designed based on the active site of the three-dimensional protein structure of human PHD2 and inhibit HIF-1α degradation [102, 103]. Moreover, FK506-binding protein 38 can reduce the stability of PHD2 protein by interacting with the N-terminal domain of PHD2, thereby accumulating HIF-1 and increasing cartilage stability [104].

The NLRP3 inflammasome (HIF-1α/NLRP3 inflammasome) is one of the inflammasome signal transduction pathways closely related to the KOA process, and inhibiting the activation of the NLRP3 inflammasome can improve synovial fibrosis in KOA. Researchers found that Agnuside (AGN), a nontoxic, natural small molecule isolated from the extract of *Vitex negundo L.,* can reduce the fibrosis of experimental KOA by inhibiting the accumulation of HIF-1α and the activation of NLRP3 inflammasomes [105]. Casticin is a compound purified from the Chinese herbal medicine Viticis Fructus. It has the effects of promoting the immune response [106], anti-inflammation [107], antioxidative stress [108] and antifibrosis [109]. Similarly, casticin reduces MIA-induced KOA by inhibiting HIF-1α/NLRP3 inflammasome activation. Therefore, casticin may be a potential treatment strategy for KOA [110].

In the recent years, magnesium-based biomedical devices have shown great potential for translation in orthopedics [111]. The use of magnesium ions (Mg^2+^) to promote the synthesis of cartilage matrix mediated by HIF-1α is a new treatment option for OA [112]. However, oxidative stress can reduce the expression of HIF-1α and enhance the inflammatory response, which may impair the efficacy of Mg^2+^ in the treatment of OA. Vitamin C is an effective antioxidant that can enhance the efficacy of Mg^2+^ in the treatment of OA [113].

In summary, there are many different ways to treat OA through the different HIF-1α pathways. Firstly, cartilage homeostasis can be strengthened through inhibiting degradation of HIF-1α in the early of OA, using compounds such as DMOG, FK506-binding protein 38, PHD inhibitors TM6008 and TM6089. However, with the progression of OA, the improving synovial fibrosis through inhibiting accumulation of HIF-1α become more important using compunds, such as Agnuside and Casticin. Besides, Mg^2+^ can be used to promote the synthesis of cartilage matrix mediated by hypoxia-inducible factor-1α.

## Targeting HIF-2α for OA therapy

HIF-2α is a regulatory factor for the expression of catabolic factors during the development of osteoarthritis. Therefore, HIF-2α inhibitors have potential therapeutic prospects for osteoarthritis. Studies have found that curcumin CMC2.24 regulates chondrocyte apoptosis and ECM homeostasis by inhibiting the NF-κB/HIF-2α pathway, thereby providing a new perspective for the treatment of OA [114]. One of the extracts of Cirsium japonicum var. maackii (CJM), apigenin inhibits HIF-2α through the NF-κB pathway, effectively blocking the expression of Prostaglandin-endoperoxide synthase 2 (COX-2), MMP3 and MMP13, and is worthy of use as a therapeutic drug for OA to block cartilage inflammation [115]. D-mannose inhibits chondrocyte ferroptosis enhanced by HIF-2α and has a chondroprotective effect on the progression of OA [116]. Icariin (ICA) is a typical flavonoid compound extracted from Epimedii Folium that may inhibit inflammatory damage by inhibiting the NF-κB/HIF-2α signaling pathway, thereby increasing chondrocyte viability [117]. Inhibition of syndecan-4 (SDC-4) induces the expression of microRNA-96-5p (miR-96-5p), targets HIF-2α 3′-UTR sequences and inhibits HIF-2α signaling in mouse cartilage tissue and chondrocytes. Therefore, this method may provide a potential new strategy to prevent the progression of osteoarthritis [118]. 4 (2′-Aminoethyl) amino-1,8-dimethylimidazo(1,2-a)quinoxaline (BMS-345541) is a selective inhibitor of the subunits of IκBα kinase (IKK). Intra-articular administration of BMS-345541 may inhibit the development of OA by downregulating NF-κB/HIF-2α signaling [119]. Studies using vectors to deliver siRNA and silence HIF-2α expression can prevent cartilage degradation in mice affected by OA [120]. In brief, there are many HIF-2α inhibitors (CMC2.24, CJM, D-mannose, ICA, SDC-4 and BMS-345541 *et. al*) have the potential therapeutic prospects in the OA diseases.

## Conclusion

HIF-1α α and HIF-2α have different functions in cartilage. The regulation of HIF-1α is crucial in maintaining cartilage homeostasis. It induces the expression of COL2A1, SOX9, GLUT1, EPO, NOS and VEGF to maintain cartilage homeostasis and inhibits the expression of COL1A1, COL1A2 and COL3A1 to prevent ECM degradation. HIF-2α is involved in a pathway that promotes osteoarthritis degradation and regulates the expression of genes related to chondrocyte hypertrophy and differentiation, such as COL10A1 and RUNX2, and the expression of genes related to ECM degradation, such as MMP9, MMP13, MMP3, ADAMTS. Many of those proteins may serve potential targets for novel therapy development. However, many research gaps still exists for further in-depth studies. For example, HIFs pathways may cross-talk with other pathways and regulation of the potential targets may thus result in serious side effects [121]. What’s more, the molecular mechanism of osteoarthritis is very complicated and many of those reported potential therapeutics still need further provement in clinical experiemnts [122]. In conclusion, OA is a dynamic change caused by the imbalance between the anabolic and catabolic of joint tissue. For osteoarthritis, increasing the accumulation and activity of HIF-1α to increase cartilage stability and inhibiting the activity of HIF-2α to reduce ECM degradation are promising therapeutic approaches.

## Data Availability

Not applicable.

## Abbreviations

ADAMTS: A disintegrin and metalloproteinase with thrombospondin motifs
AGN: Agnuside
AMPK: AMP-activated protein kinase
bHLH: Basic helix-loop-helix
CJM: Cirsium japonicum var. maackii
COL1A1: Collagen Type I Alpha 1
COL1A2: Collagen Type I Alpha 2
COL2A1: Collagen Type II Alpha 1
COL3A1: Collagen Type III Alpha 1
COL10A1: Collagen Type X Alpha 1
COX-2: Prostaglandin-endoperoxide synthase 2
DMOG: Dimethyloxaloylglycine
ECM: Extracellular matrix
EPO: Erythropoietin
FBW7: F-box and WD repeat domain-containing 7
FIH-1: Factor inhibiting HIF-1
HIF: Hypoxia-inducible factor
FasL: Fas ligand
FLS: Fibroblast-like synovial cell
HSP70: Heat shock protein 70
HRE: Hypoxia response elements
ICA: Icariin
IKK: IκBα kinase
IL: Interleukin
LncHIFCAR: Long noncoding HIF-1α co-activating RNA
LncRNA: Long noncoding RNAs
LOX: Lysyl oxidase
MAPK: Mitogen-activated protein kinase
MMP: Matrix metalloproteinase
mTOR: The mammalian target of rapamycin
NAMPT: Nicotinamide phosphoribosyltransferase
NF-κB: Nuclear factor kappa-light-chain-enhancer of activated B cells
NGF: Nerve growth factor
NLRP3: Nucleotide-binding oligomerization domain-like receptor family pyrin domain-containing 3
NOS2: Nitric oxide synthase 2
OA: Osteoarthritis
ODD: Oxygen-dependent degradation
OPN: Osteopontin
PI3K: Phosphoinositide 3 kinase
PTGS2: Prostaglandin intra peroxidase synthase 2
PHD: HIF prolyl hydroxylase
pVHL: Von Hippel−Lindau tumor suppressor protein
RUNX2: Runt-related transcription factor 2
SDC-4: Syndecan-4
SiRNA: Small interfering RNA
SOX9: SRY-Box Transcription Factor 9
TAD: Transactivation domain
TGF-β: Transforming growth factor beta
TNF: Tumor necrosis factor
TMJ: Temporomandibular joint
